# HIV-1 Vpr-Induced Proinflammatory Response and Apoptosis Are Mediated through the Sur1-Trpm4 Channel in Astrocytes

**DOI:** 10.1128/mBio.02939-20

**Published:** 2020-12-08

**Authors:** Ge Li, Tapas Makar, Volodymyr Gerzanich, Sudhakar Kalakonda, Svetlana Ivanova, Edna F. R. Pereira, Sanketh Andharvarapu, Jiantao Zhang, J. Marc Simard, Richard Y. Zhao

**Affiliations:** a Department of Pathology, University of Maryland School of Medicine, Baltimore, Maryland, USA; b Department of Neurology, University of Maryland School of Medicine, Baltimore, Maryland, USA; c Department of Neurosurgery, University of Maryland School of Medicine, Baltimore, Maryland, USA; d Department of Epidemiology and Public Health, University of Maryland School of Medicine, Baltimore, Maryland, USA; e Department of Microbiology-Immunology, University of Maryland School of Medicine, Baltimore, Maryland, USA; f Institute of Global Health, University of Maryland School of Medicine, Baltimore, Maryland, USA; g Institute of Human Virology, University of Maryland School of Medicine, Baltimore, Maryland, USA; Johns Hopkins Bloomberg School of Public Health

**Keywords:** Human immunodeficiency virus type 1 (HIV-1), HIV-associated neurocognitive disorders (HAND), viral protein R (Vpr), neuroinflammation, neurotoxicity, TLR4, TNF-α, NF-κB, Sur1-Trpm4 channel, glibenclamide, brain tissues, astrocytes, host-pathogen interactions, human immunodeficiency virus

## Abstract

Successful treatment of HIV-infected patients with combinational antiretroviral therapies (cART) can now prolong patients’ lives to nearly normal life spans. However, the new challenge faced by many of those HIV-infected patients is chronic neuroinflammation and neurotoxicity that often leads to HIV-associated neurocognitive disorders (HAND). However, the mechanism of neuropathogenesis underlying HAND, especially in those who are under cART, is not well understood. HAND is typically characterized by HIV-mediated glial neuroinflammation and neurotoxicity. However, the severity of HAND does not always correlate with HIV-1 viral load but, rather, with the extent of glial activation, suggesting that other HIV-associated factors might contribute to HAND. HIV-1 viral protein R (Vpr) could be one of those viral factors because of its association with neuroinflammation and neurotoxicity. The objective of this study was to delineate the specific roles of HIV-1 infection and Vpr in the activation of neuroinflammation and neurotoxicity, and the possible relationships with the Sur1-Trpm4 channel that contributes to neuroinflammation and neuronal death. Here, we show that HIV-1 expression correlates with activation of proinflammatory markers (TLR4, TNF-α, and NF-κB) and the Sur1-Trpm4 channel in astrocytes of HIV-infected postmortem human and transgenic Tg26 mouse brain tissues. We further show that Vpr alone activates the same set of proinflammatory markers and Sur1 in a glioblastoma SNB19 cell line that is accompanied by apoptosis. The Sur1 inhibitor glibenclamide significantly reduced Vpr-induced apoptosis. Together, our data suggest that HIV-1 Vpr-induced proinflammatory response and apoptosis are mediated at least in part through the Sur1-Trpm4 channel in astrocytes.

## INTRODUCTION

There are about 38 million people currently living with HIV/AIDS worldwide. Successful treatment with combinational antiretroviral therapies (cART) can eliminate active replicating viruses and prolong patients’ lives to nearly normal lifespans. However, the new challenge faced by many patients, especially aging patients, is chronic neuroinflammation and neurotoxicity involving the central nervous system (CNS), leading to various HIV-associated neurocognitive disorders (HAND). More than 50% of HIV-infected individuals are afflicted with HAND. While severe and progressive HAND has decreased significantly due to cART, mild-to-moderate neurocognitive impairment often persists and contributes to increased prevalence of delirium, dementia, and depression, all of which can contribute to increased rates of suicide among HIV-infected patients (1). Indeed, the risk of suicide in HIV-infected persons is significantly higher than in HIV-non-infected counterparts (2, 3). Nevertheless, the mechanism of neuropathogenesis underlying HAND is not well understood. Glial cells such as microglia or astrocytes are thought to be the primary host innate immune effectors in HIV CNS infection, which triggers proinflammatory responses and activates neurotoxic astrocytes to kill neurons (4). Thus, glia play a vital role in chronic neuroinflammation and neurotoxicity that is responsible for various manifestation of HAND, which includes not only progressive neurocognitive disorders, but also residual neurological impairment (5). Chronic HAND is characterized by glial activation, cytokine/chemokine dysregulation, and neuronal damage and loss. Interestingly, the severity of some HAND types is not directly correlated with the level of HIV replication or viral load, but rather with the level of glial activation (6), suggesting other HIV-associated factors, not the whole virus *per se*, contribute to those HAND symptoms.

HIV-1 viral protein R (Vpr) might be one of those HIV-1 proteins contributing to HAND, because: (i) in the absence of active HIV-1 viral replication under cART, Vpr can still be detected in the circulation as a soluble and free protein (7); (ii) Vpr is a transducing protein that can be taken up by glia and neurons (8); (iii) Vpr is a neurotoxin that induces neuronal apoptosis (9); (iv) Vpr binds to the HIV-1 LTR promoter and triggers viral transcription in latently infected cells (10); and (v) effects of Vpr are linked to various types of HAND (11, 12). Despite these lines of evidence indicating a prominent role of Vpr in HAND, how exactly Vpr contributes to HAND remains elusive. The objective of this study was to delineate the specific role of HIV-1 infection and Vpr in triggering host innate proinflammatory responses and their consequences. Specifically, we were interested in testing activation of neuroinflammation and neurotoxicity and, in particular, their possible association with the Sur1-Trpm4 channel that contributes to neuroinflammation and neurotoxicity (13, 14).

The Sur1 (sulfonylurea receptor 1) and Trpm4 (transient receptor potential melastatin 4) are two subunits of the Sur1-Trpm4 cation channel. The regulatory subunit Sur1, encoded by the *Abcc8* gene, is a member of the ATP-binding cassette protein superfamily, while the pore-forming subunit Trpm4 is encoded by the *Trpm4* gene. Sur1 and Trpm4 associate to form heterodimers and a functional Sur1-Trpm4 channel antagonizes calcium influx mediated by receptor-operated calcium entry (ROCE) cation channels (15). This Sur1-Trpm4 ion channel is not expressed constitutively but is transcriptionally upregulated in microglia and astrocytes (16) in responsive to various neuroinflammatory brain conditions, including traumatic brain injury (TBI) (17), subarachnoid hemorrhage (SAH) (18, 19), and neuroinflammatory conditions such as multiple sclerosis (MS)/experimental autoimmune encephalomyelitis (EAE) (13, 20). Thus, the Sur1-Trpm4 channel is a key neuroregulator involved in various neurological disorders, including brain injuries and neurodegeneration (4, 21). In this study, possible involvement of the Sur1-Trpm4 channel in HIV-1 expression and/or HIV-1 Vpr-mediated neuroinflammation and neurotoxicity was tested in postmortem brain tissue from HIV-infected subjects, brain tissues from HIV-1 transgenic 26 (Tg26) mice, and in a human glioblastoma cell line SNB19. The Tg26 mice (22) were used here because their pathological presentation resembles HAND in many respects (23) and they have clinical relevance to cART and continuous stress posed by HIV-1 and viral proteins (24).

## RESULTS

### HIV-1 infection increases expression of inflammatory markers (TLR4, TNF-α, and NF-κB) and upregulates Sur1 and Trpm4 in astrocytes of Tg26 mouse brain tissues.

**FIG 1:**
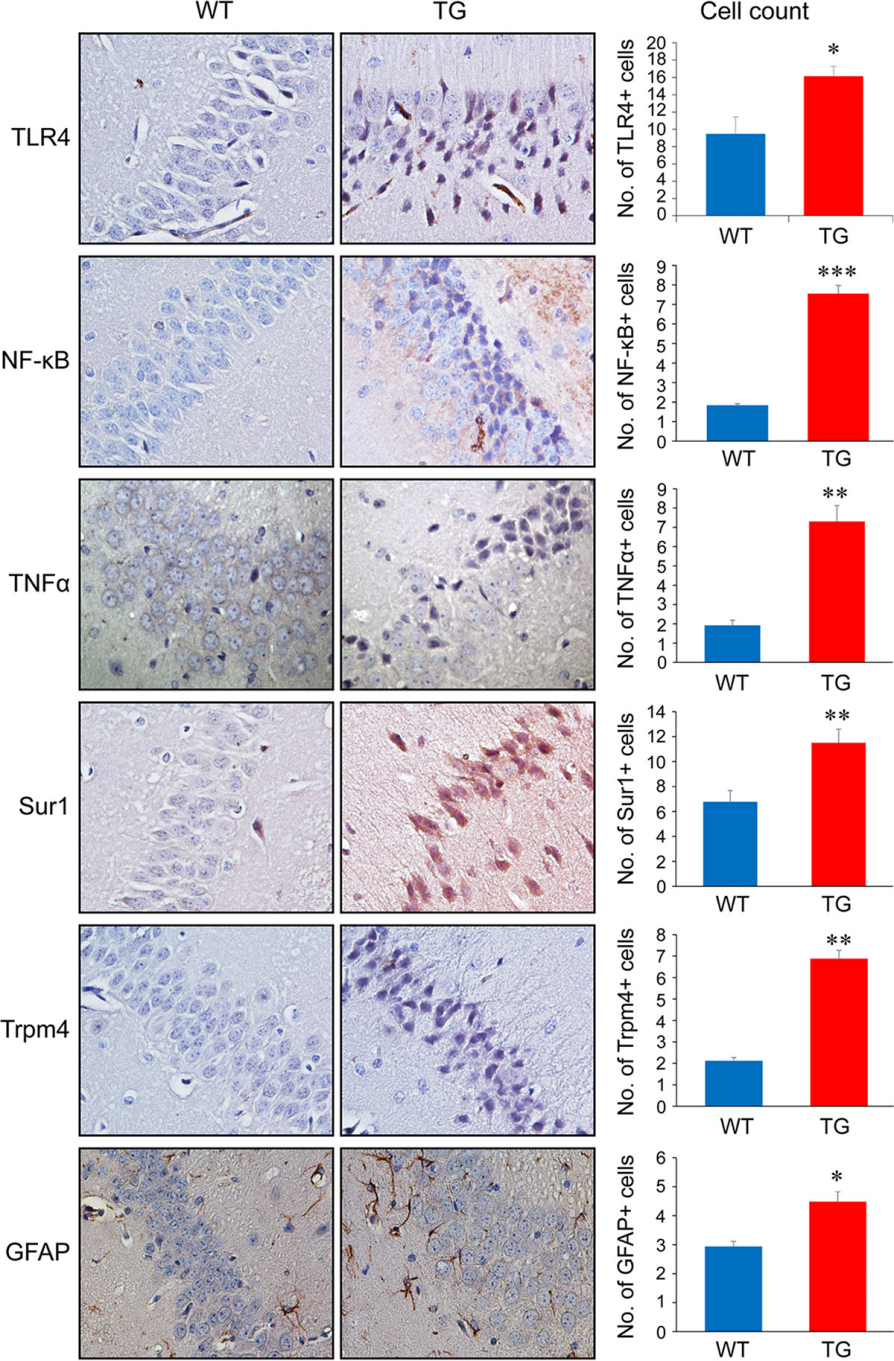
Correlation of HIV-1 expression with proinflammatory markers in the hippocampi of Tg26 mice. Each panel shows immunohistochemical staining of the hippocampal sections from the wild-type (WT) mice and Tg26 (TG) mice with protein-specific antibodies. Bar graphs show quantification of the number of immune-positive cells in hippocampal sections of the WT and TG mice. Graphs and error bars represent mean and SD, respectively, of results obtained from 2 to 3 mice/group. The total numbers of immune-positive cells counted (WT, TG) for each antibody were TLR4 (265, 1,405), NF-κB (223, 558), TNF-α (25, 480), Sur1 (1,042, 378), Trpm4 (303, 515), and GFAP (138, 332), respectively. All images are 40× magnification. Two-tailed and paired *t* test were used for statistical comparison analysis: *, *P* < 0.05; **, *P* < 0.01; ***, *P* < 0.001.

The goal of this experiment was to evaluate the impact of HIV-1 infection on neuroinflammation in the brains of Tg26 mice, which represent a cART-relevant HIV mouse model whose pathological presentation resembles many aspects of HAND (22–24). Immunohistochemistry (IHC) was used to detect host innate immune proinflammatory responses and the expression of the Sur1-Trpm4 channel. As shown in Fig. 1, expression of three proinflammatory regulators (TLR4, NF-κB, and TNF-α) was significantly higher (*, *P < *0.05; ***, *P* < 0.001) in the hippocampus of the Tg26 (TG) mice than in the wild-type (WT) control mice. Increased expression of TLR4, NF-κB, and TNF-α was accompanied by upregulation of glial fibrillary acidic protein (GFAP), a biomarker of astrocytes. GFAP is also an indicator of reactive astrogliosis near areas of active HIV-1 replication in microglial nodules (6, 25). Interestingly, expression of both Sur1 and Trpm4 proteins, which together form the Sur1-Trpm4 channel that regulates proinflammatory responses (20, 21), was also highly elevated (***, *P < *0.001).

**FIG 2:**
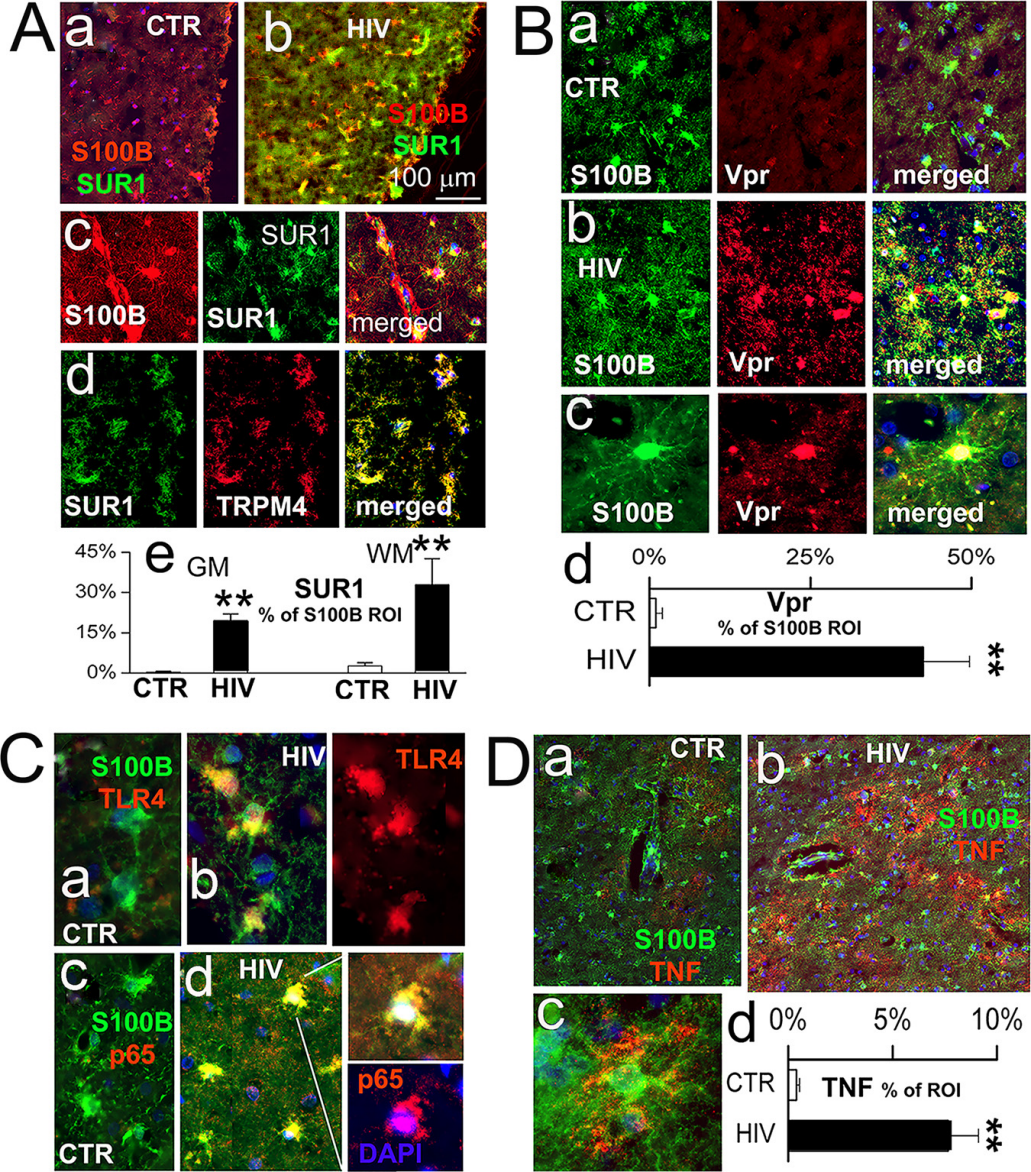
Correlation of HIV and Vpr with host innate immune proinflammatory response in astrocytes of HIV-infected postmortem human brain tissues. (A) Elevation of Sur1, where Trpm4 localizes in HIV-infected astrocytes in control (CTR) (a) HIV-infected (b) cells. Panel (c) gives an enlarged view of (b). S100B (red, left panel) shows where astrocytes are located (50), while Sur1 (green) is shown in the middle panel. Panel (d) shows colocalization of Sur1 with Trpm4. GM, gray matter; WM, white matter. (B) Vpr (red) is highly expressed in astrocytes (S100B-labeled cells, green) compared with CTR. (C): Elevation of TLR4 (a and b, red) and nuclear NF-κB (p65) (c and d, red) in HIV-infected astrocytes (S100B-labeled cells, green) compared with CTR. DAPI (blue). (D): Elevated TNF-α (red) in astrocytes (green). Graphs shown in A (e), B (d), and D (d) are astrocyte-specific expression. The S100B signals were used as the region of interest (ROI), and signals for Sur1, Vpr, and TNF-α are presented as the percentage of ROI. Note that the difference between CTR with NF-κB (p65) did not reach statistical significance. Data were quantified from brain tissues imaged from 3 HIV-negative controls and 2 HIV-positive cases (two cortical areas were used for each case). Two-tailed and unpaired *t* tests were used; **, *P* < 0.01.

To determine whether upregulation of proinflammatory markers TLR4, TNF-α, NF-κB, and Sur1 in the hippocampi of the Tg26 mice was the result of higher levels of gene transcription, reverse transcriptase quantitative PCR (RT-qPCR) was used for mRNA transcriptional analyses of the same mouse brain tissues. As expected, *gag* and the *vpr* gene transcripts were detected in the hippocampi of the TG mice but not in the hippocampi of WT mice (Fig. 2, inset). Additional testing showed that the *gag*/*vpr* expression was accompanied by overexpression of the genes that encode TLR4, TNF-α, and NF-κB, as well as the *Abcc8* gene that encodes Sur1 (Fig. 2). Note that early studies on Tg26 mice show that the abundance of HIV-1 proteins is low in tissues (24, 26). Thus, we decided to use RT-qPCR analysis here instead of Western blot analysis because HIV-1 *gag* and *vpr* genes are transcribed in Tg26 mice (26).

### Expression of HIV-1 and Vpr is accompanied by upregulation of Sur1-Trpm4 and host innate proinflammatory signaling in astrocytes of postmortem brain tissues of HIV-infected patients.

**FIG 3:**
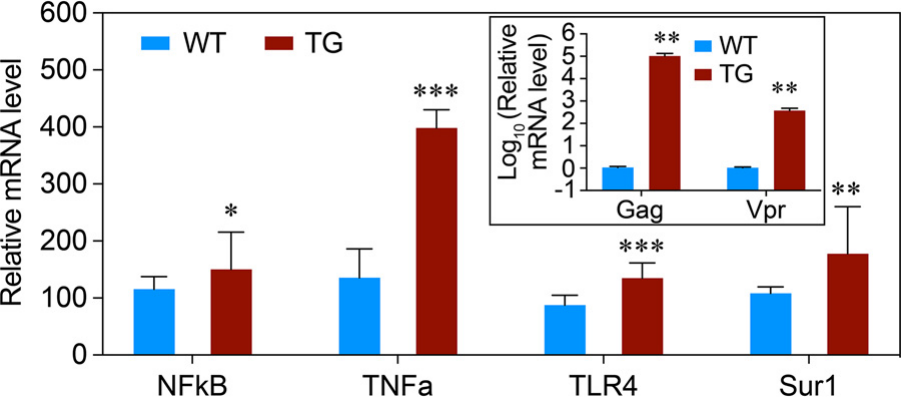
Correlation of HIV with host innate proinflammatory response in the brain tissues of Tg26 mice as measured by mRNA transcriptional analysis. Transgenic HIV-1 gene expression was confirmed by detecting the *gag* and the *vpr* gene transcription in the Tg26 mice (TG) but not in normal WT mice (inset). Graph and error bars of *TLR4*, *TNF-α*, *NF-κB*, and *Abcc8/*Sur1 represent mean and SD of mRNA transcripts. Data were analyzed by two-tailed and heteroscedatic *t* test: *, *P < *0.05; **, *P < *0.01; ***, *P* < 0.001.

We next used the same IHC method to examine whether the same inflammatory markers are also elevated in astrocytes of HIV-infected human brains. Postmortem brain tissues from two HIV-infected and three noninfected control subjects in regions of hippocampus and cerebellum were immunolabeled with the respective antibodies. Consistent with the results from the Tg26 mice, expression of Sur1 was significantly elevated (**, *P < *0.01) in hippocampus astrocytes from HIV-infected patients (Fig. 3A, b and c) compared to noninfected patients (Fig. 3A, a), as shown by quantifying cells double labeled for Sur1 and S100B, a Ca^2+^-binding peptide mainly found in astroglial cells. Furthermore, the Sur1 protein colocalized with Trpm4 (Fig. 3A, d), consistent with upregulation of the Sur1-Trpm4 channel (Fig. 3A, e). In agreement with the HIV-positive status of these brain tissues, HIV-1 Vpr protein was highly expressed in astrocytes (Fig. 3B), which also overexpressed TLR4, NF-κB (p65) (Fig. 3C), and TNF-α (Fig. 3D). Note that although the level of NF-κB (p65) increased, the difference between control (CTR) and NF-κB (p65) did not reach statistical significance. Together, these results indicated that expression of Vpr was colocalized with upregulated expression of TLR4, TNF-α, NF-κB, and Sur1-Trpm4 in astrocytes of brain tissue from HIV-infected patients.

### Expression of HIV-1 *vpr* increases expression of the same set of proinflammatory markers and Sur1 in human glioblastoma SNB19 cells.

**FIG 4:**
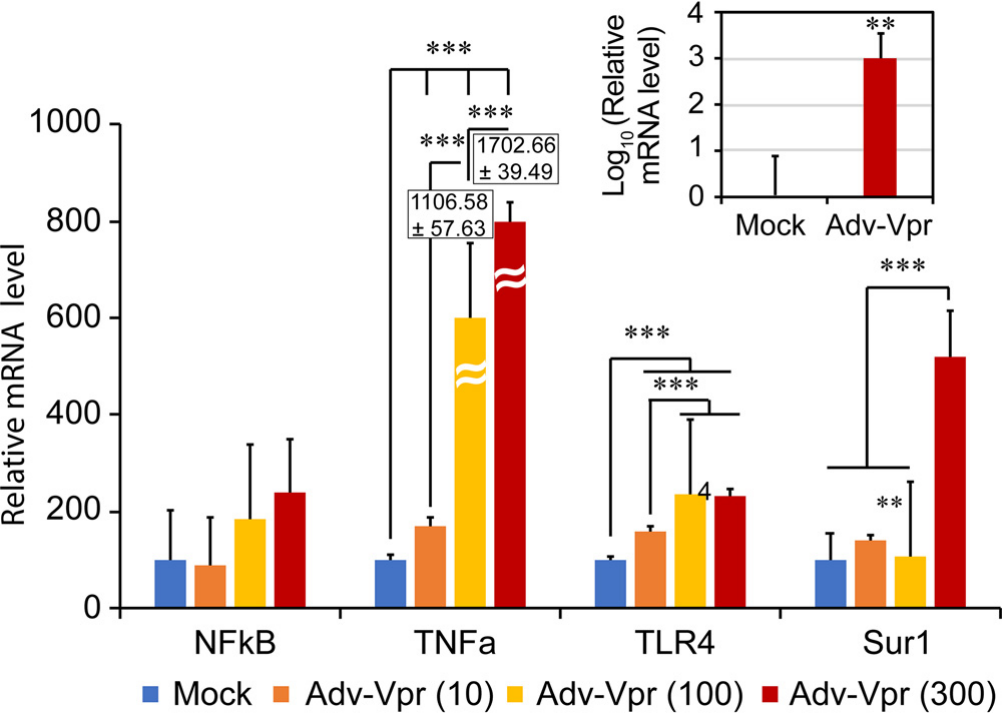
HIV-1 Vpr activates concentration-dependent gene transcription of *TLR4*, *TNF-α*, *NF-κB*, and *Abcc8* (Sur1) in human glioblastoma SNB19 cells. Adv-Vpr, adenoviral Vpr. Mock or Adv-Vpr transduced cells with MOIs of 10, 100, and 300 were collected at 24 h postinfection (hpi) when no significant death was observed. RT-qPCR was used to measure gene transcription. To avoid any potential biases caused by possible residual Vpr-induced cytotoxicity, gene transcription was measured based on equal input mRNA. The inset shows HIV-1 *vpr* gene transcription in mock- and Adv-Vpr-transduced cells. Graph and error bars represent mean and SD, respectively. One-way ANOVA followed by Tukey test was used for statistical comparison analysis; **, *P < *0.01; ***, *P < *0.001. Note that although the expression of NF-κB also showed Vpr dose-dependent increase, due to the high experimental variability, the concentration-response relationship did not reach statistical significance. Also note that both mock control and an adenovirus control were used in this experiment. The mock control was used as a calibrator to establish the baseline for comparisons. Since there were little or no significant differences between the mock control and the Adv control, only the mock control is shown in this figure.

To evaluate the contribution of HIV-1 Vpr protein to the observed upregulation of proinflammatory markers and Sur1-Trpm4 in astroglial cells, we delivered the *vpr* gene using an adenoviral (Adv) system to a glioblastoma SNB19 cell line (RRID:CVCL_0535 from NCI) with increasing multiplicity of infection (MOI). Note that the SNB19 cell line we used here is not the initial and misidentified cell line from ATCC but a confirmed cell line from NCI (27). A mock control, an Adv control, or Adv-Vpr-transduced cells were harvested at 24 h postinfection (hpi). RNA was isolated and subjected to RT-qPCR for mRNA transcriptional analysis. As shown in Fig. 4, Vpr increased, in an MOI-dependent manner, the transcription of genes encoding TLR4, TNF-α, and the Sur1-encoding *Abcc8* gene. Transcriptional expression of the gene encoding NF-κB was also increased with the increase of Vpr concentration; however, due to high experimental variability, the MOI-response relationship did not reach statistical significance. Note that the mock control was used as a calibrator to establish the baseline for comparisons. Since there were little or no significant differences between the mock control and the Adv control, only the mock control is shown in Fig. 4.

### Inhibition of Vpr-induced apoptosis by the Sur1 inhibitor glibenclamide in SNB19 cells.

**FIG 5:**
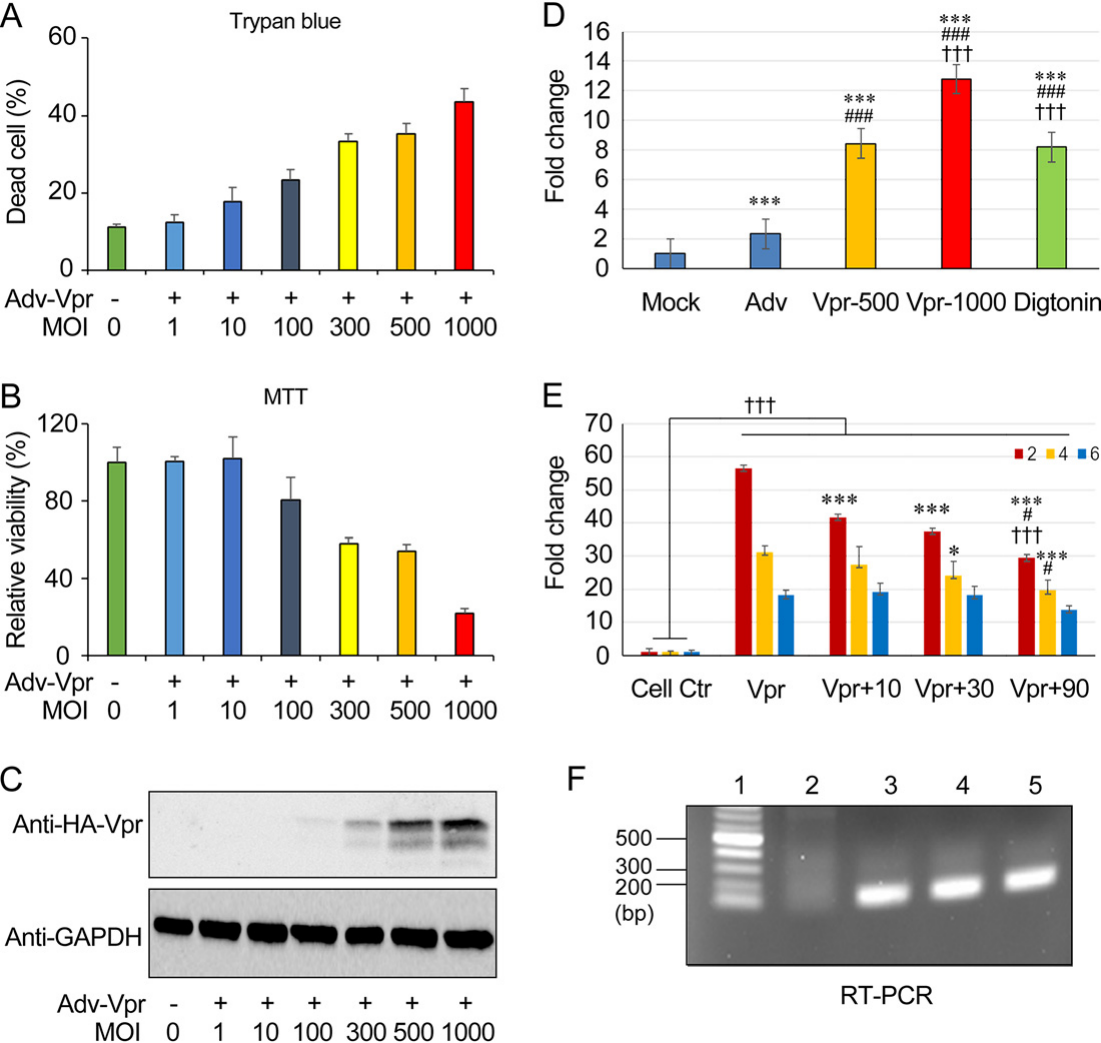
Inhibition of HIV-1 Vpr-induced apoptosis by Sur1 inhibitor glibenclamide in human glial SNB19 cells. HIV-1 Vpr-induced cell death and apoptosis were measured with different MOIs at 5 dpi. (A) Cell death assessed by trypan blue straining. (B) Cell viability assessed using the MTT assay. (C) Vpr protein production confirmed by Western blotting. Note that it was difficult to detect Vpr protein at low MOI. (D) Titer-dependent induction of Vpr-induced early apoptosis, which was measured by a RealTime Annex V assay (Promega) at 6 hpi. Mock, nothing added; Adv, adenovirus control, MOI, 1,000; Digtonin, an apoptosis-inducing agent as a positive control. Graph and error bars represent mean and SD, respectively, of results from at least three independent cultures. One-way ANOVA followed by Tukey multigroup comparison were used. Treatment: *P* < 0.001; versus mock: ***, *P* < 0.001; versus Adv: ###, *P* < 0.001; versus Vpr-500, †††, *P* < 0.001. (E) Concentration-dependent inhibition of Vpr-induced apoptosis by glibenclamide (10, 30, and 90 μM) at 2, 4, and 6 hpi. Two-way ANOVA followed by Tukey multigroup comparison were used. Treatment: *P* < 0.001; Time: *P* < 0.001; Treatment × Time: *P* < 0.001; versus Vpr: *, *P* < 0.05, ***, *P* < 0.001; versus 30 μM glibenclamide: #, *P* < 0.05; versus 10 μM glibenclamide: †††, *P* < 0.001. An MOI of 1,000 was used for both the Adv control and Adv-Vpr. (F) The *vpr* gene transcription measured by RT-PCR at 6 hpi. Lanes: 1, DNA ladder; 2, Adv control; 3, Adv-Vpr; 4, Adv-Vpr + glibenclamide; 5, An Adv-Vpr positive control. SNB19 cells were infected with Adv-Vpr in a 96-well microtiter plate with an MOI of 1,000 for both the Adv control and Adv-Vpr. Graph and error bars represent mean and SD, respectively, of results from at least three independent cultures.

We next tested whether Vpr confers neurotoxicity in the human glioblastoma SNB19 cell line that has a glial cell origin. Cells were transduced by Adv-Vpr with increasing MOIs, and cell viability was determined by 5 days postinfection (dpi) (Fig. 5). While the increase of MOI of the Adv control did not cause significant cell death, a clear concentration-dependent cell killing was shown as indicated by the trypan blue assay (Fig. 5A), which was accompanied by a concentration-dependent decrease of cell viability by the MTT (3-[4,5-dimethyl-2-thiazolyl]-2,5-diphenyl-2H-tetrazolium bromide) assay (Fig. 5B) and a concentration-dependent increase of altered and deleterious cell morphologic changes (data not shown). Western blot analysis of Adv-Vpr-transfected cell cultures suggested proper production of Vpr proteins (Fig. 5C). A RealTime Annex V assay (Promega) was used to elucidate whether Vpr-induced cell death had an apoptotic component. As shown in Fig. 5D, positive-control digitonin indicated proper induction of apoptosis. In mock- or Adv-infected cultures that were not exposed to Vpr or any apoptosis-inducing stimulus, there was no significant cell death. In contrast, Adv-Vpr-transduced cells showed a dose-dependent increase in apoptosis that was significantly higher than the controls.

Since Vpr increases the expression of Sur1 (Fig. 4), we tested whether a Sur1-specific inhibitor, glibenclamide, could suppress the Vpr effect. Vpr-induced apoptosis was measured the same way as shown in Fig. 5D, except that glibenclamide was added at increasing concentrations from 10 to 30 to 90 μM. A concentration-dependent inhibition of Vpr-induced apoptosis by glibenclamide was observed at 2, 4, and 6 hpi (Fig. 5E). Statistical analysis shows these Vpr concentration-dependent decreases were significant. RT-qPCR analysis indicated that the *vpr* gene was properly expressed (Fig. 5F). Together, these data suggest that Vpr induces neurotoxicity via apoptosis in SNB19 cell cultures, which can be alleviated at least in part by the Sur1 inhibitor glibenclamide in a concentration-dependent manner.

## DISCUSSION

Results of this study show the correlation between HIV-1 expression and activation of the proinflammatory markers TLR4, TNF-α, and NF-κB in astrocytes of HIV-transgenic Tg26 mouse (Fig. 1 and 2) and HIV-infected postmortem human brain tissues (Fig. 3). These data suggest that HIV-1 infection triggers proinflammatory responses of the CNS. In addition, the activation effect of HIV-infection is at least partly due to the presence of the HIV-1 Vpr protein, as expression of Vpr alone activates the same set of proinflammatory markers in human glial cells and Tg26 mouse brain tissues (Fig. 4). Furthermore, the production of Adv-Vpr induces apoptosis in SNB19 cells (Fig. 5), consistent with Vpr inducing neurotoxicity via apoptosis. Importantly, HIV-1 infection or Vpr production was also correlated with the upregulation of the Sur1-Trpm4 channel (Fig. 1 to 4), and a Sur1 inhibitor glibenclamide inhibited Vpr-induced apoptosis in a concentration-dependent manner in SNB19 cells (Fig. 5). Together, these data suggest that HIV-1 Vpr-induced proinflammatory response and apoptotic cell death are mediated, at least in part, through the Sur1-Trpm4 channel in astrocytes. This is the first study to demonstrate that, via a Sur1-Trmp4-dependent mechanism, HIV-1 Vpr contributes to the increased expression of the inflammatory markers TLR4, TNF-α, and NF-κB detected in hippocampal astrocytes of HIV-transgenic Tg26 mouse and in brain tissues from HIV-infected patients.

HAND includes a range of neurological disorders of various severity caused by HIV-1. HIV-1 infects the CNS as early as 8 h after initial infection (28). In the human brain, HIV targets primarily the subcortical areas, including hippocampus and cerebellum, where it infects glial cells, including astrocytes, which represent the most abundant type of glial cells, and microglia, which are resident macrophage-like cells in the CNS. HIV-1 infection of glial cells triggers host proinflammatory immune responses by stimulating reactive astrocytes that release cytokines/chemokines and neurotoxic factors, all of which contribute to neurodegeneration, compromise neuronal function, and induce neurocognitive impairment (29). While cART is effective in eliminating active replicating viruses, it has little or no effect on HIV-1 proviruses that reside in quiescent astrocytes, which therefore become latent reservoirs in patients who otherwise have no detectable or residual HIV-1 under cART (30). In the absence of active viral replication, chronic HIV infection caused by low levels of viral activities or secretion of neurotoxins of both host and viral origins continue to cause persistent neuroinflammation with neurotoxicity, leading to chronic HAND (4). Consequently, the severity of some HAND does not always directly correlate with the level of HIV replication or viral load, but rather with lasting glial activation (6), suggesting that other HIV-associated factors but not the whole virus *per se* contribute to those cases of HAND. Results of this study provide strong support that HIV-1 Vpr is one of those HIV-associated proteins contributing to HAND, at least in part through the Sur1-Trpm4 channel.

Vpr exerts its effects on the CNS both as an intracellular and an extracellular protein. As an intracellular protein, it is involved in establishing and sustaining CNS infection, as its function is required for HIV-1 replication in nondividing monocyte-derived macrophages, a key lineage of cells involved in HIV-1 neuro-invasion (31). Vpr can also be found as an extracellular and free circulating protein in blood, cerebrospinal fluid, and CNS-associated cells (7, 12, 32–37), where Vpr reactivates HIV replication from latency (10, 32, 33, 36) (for detailed reviews, see reference 7). Vpr triggers proinflammatory reactions (7, 12, 35) and promotes astroglial secretion of the proinflammatory cytokine interleukin 6 (IL-6) (38). Vpr is also a neurotoxin that induces apoptosis in astrocytes (35), neurons (9), and other cells in the CNS (39). Consequently, Vpr causes neurodegeneration (9), synaptic loss, and neurocognitive impairment (40) that link to various types of HAND (11, 12). Consistent with the literature, our results support a specific role of Vpr-induced neuroinflammation and neurotoxicity in HAND.

In this study, the expression of neuroinflammatory markers and Sur1 was examined not only in postmortem brain tissue from HIV-infected patients and noninfected patients, but also in brain tissue from Tg26 mice. These transgenic mice lack active HIV replication but develop HAND due to continuous stress from viral proteins and, as such, are considered to be a relevant model of HIV-1-associated comorbidities present in cART-treated HIV patients (22–24). In addition, the effects of Vpr on expression of inflammatory markers and Sur1 were investigated in the astrocytic cell line SNB19 in the presence and absence of glibenclamide. Results presented here reveal that: (i) markers of inflammation and Sur1 are overexpressed in brain tissue of HIV-infected patients and Tg26 mice; (ii) Vpr alone increases expression of Sur1 and inflammatory markers in SNB19 cells; and (iii) the Sur1-Trpm4 inhibitor glibenclamide suppresses Vpr-induced apoptosis in a Vpr concentration-dependent manner in SNB19 cultures.

One of the more significant findings of this study is that we discovered a link between Vpr and the Sur1-Trpm4 channel. The association of Sur1 with Trpm4 generates functional Sur1-Trpm4 ion channels that act as nonselective, cation-permeable channels (41). These channels are unique in that they are expressed in the CNS in response to injuries (reviewed in reference 42). Numerous studies have demonstrated that increased expression of functional Sur1-Trpm4 channels results in excessive Na^+^ influx that leads to cell swelling, which, in turn, contributes to necrotic cell death and neuroinflammation in preclinical models of TBI, SAH, and MS/EAE (reviewed in reference 16). In addition, pharmacological block of Sur1-Trpm4 with glibenclamide attenuates neuroinflammation and neurodegeneration and improves cognitive functions in those animal models. Thus, the Sur1-Trpm4 channel is a key neuro-regulator involved in various neurological disorders, including brain injuries and neurodegeneration (4, 21).

Furthermore, the link between Vpr and the Sur1-Trpm4 channel is functionally relevant because Vpr has been shown to activate the nuclear factor of activated T cells (NFAT) through Ca^2+^ influx in neuronal cells (10, 12), and Sur1-Trpm4 activation has also been shown to increase nuclear translocation of NFAT in the N9 murine microglial cell line (21). Therefore, it is possible that Sur1-Trpm4 channel-induced neuropathological effects are responsible for the Vpr effects, or are part of the collective induction of Vpr-induced HAND. If confirmed, this finding would be clinically significant because the Sur1-Trpm4 channel has shown to be a key drug target of various neuroinflammatory conditions (13, 17–20), and pharmacologic inhibition of this channel by the repurposed and FDA-approved drug glibenclamide significantly improved clinical outcomes of those neurologic disorders (14, 20, 43–45). Indeed, the Sur1 inhibitor glibenclamide suppressed Vpr-induced apoptosis in a concentration-dependent manner (Fig. 5E and F).

In conclusion, the results presented here suggest that Vpr, acting via a Sur1-Trpm4 channel-dependent mechanism, contributes to the pathophysiology of HAND. This finding provides a novel mechanistic framework for future studies aimed at determining whether HAND can be effectively treated with the FDA-approved Sur1 inhibitory drug glibenclamide, which has already been shown to be an effective therapeutic intervention for various neuroinflammatory disorders (14, 20, 43–45).

## MATERIALS AND METHODS

### Cell culture and tissues.

SNB19 (RRID:CVCL_0535 from NCI), provided by H.L. Tang (27, 46), is a human glioblastoma cell line that develops from glial cells. It was maintained in RPMI 1640 medium supplemented with 10% fetal bovine serum (FBS, Invitrogen) and 100 units/ml penicillin plus 100 μg/ml streptomycin. Note that the SNB19 cell line we used in this study is not the initial and misidentified cell line from ATCC but rather a confirmed cell line from NCI (27). Postmortem human brain tissues from two HIV-infected and three noninfected individuals were from the University of Maryland Baltimore Brain and Tissue Bank. Mouse brain tissues were harvested from HIV-transgenic Tg26 FVB/N mice, which contain a partial pNL4-3 HIV-1 genome of 7.4-kb that encompasses most of the *gag* and *pol* genes, including the *vpr* gene, as has been described previously (47). FVB/N mice were used as control animals because this is the genetic background of the Tg26 mice. Adult (16 weeks old) male and female mice were used in this study. All animal experimental procedures were approved by the University of Maryland School of Medicine Institutional Animal Care and Use Committee (IACUC) and were conducted in full accordance with the National Institutes of Health Guide for the Care and Use of Laboratory Animals. The hippocampal and cortex regions of the brain sections were used for the IHC staining.

### Adenoviral constructs and cell transduction.

An HA-tagged Adv-Vpr construct was generated in this laboratory (48, 49) by use of the AdEasy adenoviral vector system (catalog number 240009; Stratagene, La Jolla, CA). The viral titer of the Adv control was 2 × 10^9^ plaque-forming unite (PFU)/ml, and the Adv-Vpr titer was 1 × 10^9^ PFU/ml, which were determined by an enzyme-linked immunosorbent assay (ELISA) Adeno-X Rapid Titer kit (catalog number 631028, TaKaRa) that detects the adenoviral hexon surface antigen. For Adv transduction, SNB19 cells at the density of 1 × 10^4^/well in 96-well plates were seeded and incubated at 37 C°/5% CO2 overnight to allow the cells to attach to the wells. The second day, SNB19 cells were transduced with the Adv control or Adv-Vpr with the MOI as indicated in each experiment. The Adv-transduced cells were incubated at 37°C with gentle agitation. The cells were collected at the indicated times for analyses.

### Immunostaining of brain tissues.

Brains of Tg26 and WT control mice were fixed with formalin and embedded in paraffin. Paraffin-embedded mouse and human brain blocks containing the hippocampus and the cerebellar cortex regions were cut in a microtome for subsequent IHC staining using VECTASTAIN ABC kits (Vector Laboratories, Burlingame, CA). Paraffin-embedded mouse (7 μm thickness) and human brain sections (12 μm thickness) were deparaffinized and rehydrated by passing through xylene and then alcohol dilutions (100%, 95%, and 70%). After antigen retrieval, samples were incubated in blocking solution (90% Tris-buffered saline with Tween 20 [TBST] and 10% fetal bovine serum [FBS]) for 1 h, in primary antibody solutions for overnight, in secondary antibody solutions for 45 min, in avidin-biotin complex (ABC) for 30 min, and, finally, in 3,3′-diaminobenzidyne (DAB) solution until the expected color change was seen. The antibodies used in this study for IHC and Western blot analyses are listed in Table 1.

**TABLE 1:** List of primary antibodies used in IHC and Western blot analyses

Name	Poly/mono-clonal	Vendor	Catalog no.	Dilution
Mouse				
TLR4	Polyclonal	Novus Biologicals	NB100-56580	1:200
NF-κB	Polyclonal	Cell Signaling Technology, Boston, MA, USA	8242	1:200
TNFα	Polyclonal	Santa Cruz Biotechnology, Santa Cruz, CA, USA	52B83	1:500
Sur1	Polyclonal	Custom made^*a*^	NA	1:250
Trmp4	Polyclonal	Sigma-Aldrich	ABN418	1:200
GFAP	Polyclonal	EMD Millipore, Billerica, MA, USA	AB5541	1:100
Human				
Sur1	Polyclonal	Custom made^*a*^	NA	1:500
Trpm4	Polyclonal	Custom made^*a*^	NA	1:500
S100B	Monoclonal	Abcam	Ab7852	1:200
TLR4	Monoclonal	Abcam	Ab7862	1:100
NF-κB (p65)	Polyclonal	Santa Cruz Biotechnology	Sc-372	1:200
TNF-α	Polyclonal	Santa Cruz Biotechnology	Sc-1350	1:100
HIV-1 Vpr	Polyclonal	NIH AIDS Reagent Program	3951	1:100
HA	Monoclonal	Sigma	H-9658	1:1,000

a Use of these custom-made antibodies have been described previously (51); NA, not applicable.

After the staining step, slides were examined under a light microscope at the magnification of 40×. Two to four slides were used for cell counts in each group. Images were taken from both the cortex and the hippocampus areas. Five images were taken from each cortex, whereas whole cells of hippocampus were analyzed. The cells stained with the DAB substrate were taken as positive cells. Statistical Bonferroni's multiple-comparison post test was used for one-way ANOVA using Prism software (GraphPad, San Diego, CA). Statistical significance was accepted at the 95% confidence level (*P* < 0.05).

### Real-time RT-qPCR.

Total RNA was extracted from SNB19 cells or snap-frozen mouse brain tissue by using TRIzol reagent (Life Technologies) according to the manufacturer’s protocol. For snap-frozen mouse brain tissue, tissues were first homogenized by Precellys Evolution tissue homogenizer (Bertin Technologies) and then total RNA was extracted using TRIzol reagent. The RNA pellet was resuspended in RNase-free distilled water and stored at −80°C. Extracted RNA (500 ng) was used for real-time RT-qPCR analysis using iTaq Universal SYBR Green one-step kit (Bio-Rad) according to the manufacturer’s instructions. The primer sequences targeting genes of interest are listed in Table 2. Gene amplification in a Bio-Rad CFX96 RT-PCR system involved reverse transcription reaction at 50°C for 10 min, activation and DNA denaturation at 95°C for 1 min followed by 40 amplification cycles of 95°C for 15 s and 60°C for 30 s. Fold change in mRNA expression was quantified by calculating the threshold cycle (2^-ΔΔCT^) value, with glyceraldehyde-3-phosphate dehydrogenase (GAPDH) mRNA as an endogenous control.

**TABLE 2:** Primer sequences used in this study

Gene	Species	Forward primer	Reverse primer
*Gag*	HIV-1	CATGTTTTCAGCATTATCAGAAGGA	TGCTTGATGTCCCCCCACT
*Vpr*	HIV-1	GATACTTGGGCAGGAGTGGA	TGGCTCCATTTCTTGCTCTC
*Tlr4*	Mouse	AGCTTCTCCAATTTTTCAGAACTTC	TGAGAGGTGGTGTAAGCCATGC
*Nfkb*	Mouse	GAAATTCCTGATCCAGACAAAAAC	ATCACTTCAATGGCCTCTGTGTAG
*Tnfa*	Mouse	ATGAGCACAGAAAGCATGA	AGTAGACAGAAGAGCGTGGT
*Sur1*	Mouse	GCCAGCTCTTTGAGCATTGG	AGGCCCTGAGACGGTTCTG
*Trpm4*	Mouse	TGTTGCTCAACCTGCTCATC	GCTGTGCCTTCCAGTAGAGG
*Gapdh*	Mouse	GTTGTCTCCTGCGACTTCA	GGTGGTCCAGGGTTTCTTA
*NF-KB*	Human	CACTGCTCAGGTCCACTGTC	CTGTCACTATCCCGGAGTTCA
*TNFA*	Human	CGAGTGACAAGCCTGTAGC	GGTGTGGGTGAGGAGCACAT
*TLR4*	Human	TGGAAGTTGAACGAATGGAATG	ACCAGAACTGCTACAACAGATACT
*SUR1*	Human	ACTGGATGGTGAGGAACCTG	TGGATCTGGATCTTCCCTTG
*GAPDH*	Human	TGCACCACCAACTGCTTAG	AGTAGAGGCAGGGATGATGTTC

### Measurement of HIV-1 Vpr-induced apoptosis.

Cellular apoptosis was measured by a RealTime-Glo Annexin V Apoptosis and Necrosis assay kit (Promega) according to the manufacturer’s instructions. Briefly, 1 × 10^4^ cells/well were seeded into a 96-well plate and cultured at 37°C/5% CO_2_ overnight. After viral transduction with the Adv control or Adv-Vpr, the detection reagent (which included Annexin V NanoBiT substrate, CaCl_2_, Necrosis Detection Reagent, Annexin V-SmBiT, and Annexin V-LgBiT) was added into each well of a 96-well plate. Following incubation at 37°C/5% CO_2_, at the indicated time frame the luminescence (relative light units [RLU]) and fluorescence (RFU, 485nm_Ex_/520–30nm_Em_) were measured for apoptosis at different time points with a SYNERGY-H1 microplate reader. A stock solution of glibenclamide was prepared by placing 25 mg glibenclamide (number G2539; meets USP testing; Sigma-Aldrich) into 10 ml dimethyl sulfoxide (DMSO).

## References

[1] Patel S, Parikh NU, Aalinkeel R, Reynolds JL, Dmello R, Schwartz SA, Mahajan SD. 2018. United States national trends in mortality, length of stay (LOS) and associated costs of cognitive impairment in HIV population from 2005 to 2014. AIDS Behav 22:3198–3208. doi:10.1007/s10461-018-2128-z. 29705930

[2] So-Armah K, Gupta SK, Kundu S, Stewart JC, Goulet JL, Butt AA, Sico JJ, Marconi VC, Crystal S, Rodriguez-Barradas MC, Budoff M, Gibert CL, Chang CC, Bedimo R, Freiberg MS. 2019. Depression and all-cause mortality risk in HIV-infected and HIV-uninfected US veterans: a cohort study. HIV Med 20:317–329. doi:10.1111/hiv.12726. 30924577 PMC6459698

[3] Rodriguez VJ, Mandell LN, Babayigit S, Manohar RR, Weiss SM, Jones DL. 2018. Correlates of suicidal ideation during pregnancy and postpartum among women living with HIV in rural South Africa. AIDS Behav 22:3188–3197. doi:10.1007/s10461-018-2153-y. 29752621 PMC6230517

[4] Clarke LE, Liddelow SA, Chakraborty C, Munch AE, Heiman M, Barres BA. 2018. Normal aging induces A1-like astrocyte reactivity. Proc Natl Acad Sci U S A 115:E1896–E1905. doi:10.1073/pnas.1800165115. 29437957 PMC5828643

[5] Clifford DB, Ances BM. 2013. HIV-associated neurocognitive disorder. Lancet Infect Dis 13:976–986. doi:10.1016/S1473-3099(13)70269-X. 24156898 PMC4108270

[6] Tavazzi E, Morrison D, Sullivan P, Morgello S, Fischer T. 2014. Brain inflammation is a common feature of HIV-infected patients without HIV encephalitis or productive brain infection. Curr HIV Res 12:97–110. doi:10.2174/1570162x12666140526114956. 24862332 PMC4152918

[7] Ferrucci A, Nonnemacher MR, Wigdahl B. 2011. Human immunodeficiency virus viral protein R as an extracellular protein in neuropathogenesis. Adv Virus Res 81:165–199. doi:10.1016/B978-0-12-385885-6.00010-9. 22094081 PMC3731056

[8] Sherman MP, Schubert U, Williams SA, de Noronha CM, Kreisberg JF, Henklein P, Greene WC. 2002. HIV-1 Vpr displays natural protein-transducing properties: implications for viral pathogenesis. Virology 302:95–105. doi:10.1006/viro.2002.1576. 12429519

[9] Jones GJ, Barsby NL, Cohen EA, Holden J, Harris K, Dickie P, Jhamandas J, Power C. 2007. HIV-1 Vpr causes neuronal apoptosis and in vivo neurodegeneration. J Neurosci 27:3703–3711. doi:10.1523/JNEUROSCI.5522-06.2007. 17409234 PMC6672409

[10] Hohne K, Businger R, van Nuffel A, Bolduan S, Koppensteiner H, Baeyens A, Vermeire J, Malatinkova E, Verhasselt B, Schindler M. 2016. Virion encapsidated HIV-1 Vpr induces NFAT to prime non-activated T cells for productive infection. Open Biol 6:160046. doi:10.1098/rsob.160046. 27383627 PMC4967821

[11] Power C, Hui E, Vivithanaporn P, Acharjee S, Polyak M. 2012. Delineating HIV-associated neurocognitive disorders using transgenic models: the neuropathogenic actions of Vpr. J Neuroimmune Pharmacol 7:319–331. doi:10.1007/s11481-011-9310-7. 21918813

[12] Rom I, Deshmane SL, Mukerjee R, Khalili K, Amini S, Sawaya BE. 2009. HIV-1 Vpr deregulates calcium secretion in neural cells. Brain Res 1275:81–86. doi:10.1016/j.brainres.2009.03.024. 19328187 PMC2692350

[13] Tosun C, Kurland DB, Mehta R, Castellani RJ, deJong JL, Kwon MS, Woo SK, Gerzanich V, Simard JM. 2013. Inhibition of the Sur1-Trpm4 channel reduces neuroinflammation and cognitive impairment in subarachnoid hemorrhage. Stroke 44:3522–3528. doi:10.1161/STROKEAHA.113.002904. 24114458 PMC3894855

[14] Makar TK, Gerzanich V, Nimmagadda VK, Jain R, Lam K, Mubariz F, Trisler D, Ivanova S, Woo SK, Kwon MS, Bryan J, Bever CT, Simard JM. 2015. Silencing of Abcc8 or inhibition of newly upregulated Sur1-Trpm4 reduce inflammation and disease progression in experimental autoimmune encephalomyelitis. J Neuroinflammation 12:210. doi:10.1186/s12974-015-0432-3. 26581714 PMC4652344

[15] Woo SK, Kwon MS, Ivanov A, Gerzanich V, Simard JM. 2013. The sulfonylurea receptor 1 (Sur1)-transient receptor potential melastatin 4 (Trpm4) channel. J Biol Chem 288:3655–3667. doi:10.1074/jbc.M112.428219. 23255597 PMC3561583

[16] Simard JM, Woo SK, Schwartzbauer GT, Gerzanich V. 2012. Sulfonylurea receptor 1 in central nervous system injury: a focused review. J Cereb Blood Flow Metab 32:1699–1717. doi:10.1038/jcbfm.2012.91. 22714048 PMC3434627

[17] Zweckberger K, Hackenberg K, Jung CS, Hertle DN, Kiening KL, Unterberg AW, Sakowitz OW. 2014. Glibenclamide reduces secondary brain damage after experimental traumatic brain injury. Neuroscience 272:199–206. doi:10.1016/j.neuroscience.2014.04.040. 24792709

[18] Walcott BP, Kahle KT, Simard JM. 2012. Novel treatment targets for cerebral edema. Neurotherapeutics 9:65–72. doi:10.1007/s13311-011-0087-4. 22125096 PMC3271162

[19] Martínez-Valverde T, Vidal-Jorge M, Martínez-Saez E, Castro L, Arikan F, Cordero E, Rădoi A, Poca M-A, Simard JM, Sahuquillo J. 2015. Sulfonylurea receptor 1 in humans with post-traumatic brain contusions. J Neurotrauma 32:1478–1487. doi:10.1089/neu.2014.3706. 26398596 PMC4589328

[20] Gerzanich V, Makar TK, Guda PR, Kwon MS, Stokum JA, Woo SK, Ivanova S, Ivanov A, Mehta RI, Morris AB, Bryan J, Bever CT, Simard JM. 2017. Salutary effects of glibenclamide during the chronic phase of murine experimental autoimmune encephalomyelitis. J Neuroinflammation 14:177. doi:10.1186/s12974-017-0953-z. 28865458 PMC5581426

[21] Kurland DB, Gerzanich V, Karimy JK, Woo SK, Vennekens R, Freichel M, Nilius B, Bryan J, Simard JM. 2016. The Sur1-Trpm4 channel regulates NOS2 transcription in TLR4-activated microglia. J Neuroinflammation 13:130. doi:10.1186/s12974-016-0599-2. 27246103 PMC4888589

[22] Dickie P, Felser J, Eckhaus M, Bryant J, Silver J, Marinos N, Notkins AL. 1991. HIV-associated nephropathy in transgenic mice expressing HIV-1 igenes. Virology 185:109–119. doi:10.1016/0042-6822(91)90759-5. 1926769

[23] Putatunda R, Zhang Y, Li F, Fagan PR, Zhao H, Ramirez SH, Pratico D, Barbe MF, Hu W. 2019. Sex-specific neurogenic deficits and neurocognitive disorders in middle-aged HIV-1 Tg26 transgenic mice. Brain Behav Immun 80:488–499. doi:10.1016/j.bbi.2019.04.029. 30999016 PMC6660421

[24] Putatunda R, Zhang Y, Li F, Yang XF, Barbe MF, Hu W. 2018. Adult neurogenic deficits in HIV-1 Tg26 transgenic mice. J Neuroinflammation 15:287. doi:10.1186/s12974-018-1322-2. 30314515 PMC6182864

[25] Sabri F, Titanji K, De Milito A, Chiodi F. 2003. Astrocyte activation and apoptosis: their roles in the neuropathology of HIV infection. Brain Pathol 13:84–94. doi:10.1111/j.1750-3639.2003.tb00009.x. 12580548 PMC8095843

[26] Dickie P, Roberts A, Uwiera R, Witmer J, Sharma K, Kopp JB. 2004. Focal glomerulosclerosis in proviral and c-fms transgenic mice links Vpr expression to HIV-associated nephropathy. Virology 322:69–81. doi:10.1016/j.virol.2004.01.026. 15063118

[27] Tang H, Hammack C, Ogden SC, Wen Z, Qian X, Li Y, Yao B, Shin J, Zhang F, Lee EM, Christian KM, Didier RA, Jin P, Song H, Ming GL. 2016. Zika virus infects human cortical neural progenitors and attenuates their growth. Cell Stem Cell 18:587–590. doi:10.1016/j.stem.2016.02.016. 26952870 PMC5299540

[28] Atluri VS, Hidalgo M, Samikkannu T, Kurapati KR, Jayant RD, Sagar V, Nair MP. 2015. Effect of human immunodeficiency virus on blood-brain barrier integrity and function: an update. Front Cell Neurosci 9:212. doi:10.3389/fncel.2015.00212. 26113810 PMC4461820

[29] Hong S, Banks WA. 2015. Role of the immune system in HIV-associated neuroinflammation and neurocognitive implications. Brain Behav Immun 45:1–12. doi:10.1016/j.bbi.2014.10.008. 25449672 PMC4342286

[30] Churchill M, Nath A. 2013. Where does HIV hide? A focus on the central nervous system. Curr Opin HIV AIDS 8:165–169. doi:10.1097/COH.0b013e32835fc601. 23429501 PMC5241183

[31] Connor RI, Chen BK, Choe S, Landau NR. 1995. Vpr is required for efficient replication of human immunodeficiency virus type-1 in mononuclear iphagocytes. Virology 206:935–944. doi:10.1006/viro.1995.1016. 7531918

[32] Hoshino S, Konishi M, Mori M, Shimura M, Nishitani C, Kuroki Y, Koyanagi Y, Kano S, Itabe H, Ishizaka Y. 2010. HIV-1 Vpr induces TLR4/MyD88-mediated IL-6 production and reactivates viral production from latency. J Leukoc Biol 87:1133–1143. doi:10.1189/jlb.0809547. 20145198

[33] Levy DN, Refaeli Y, MacGregor RR, Weiner DB. 1994. Serum Vpr regulates productive infection and latency of human immunodeficiency virus type 1. Proc Natl Acad Sci U S A 91:10873–10877. doi:10.1073/pnas.91.23.10873. 7971975 PMC45128

[34] Hoshino S, Sun B, Konishi M, Shimura M, Segawa T, Hagiwara Y, Koyanagi Y, Iwamoto A, Mimaya J, Terunuma H, Kano S, Ishizaka Y. 2007. Vpr in plasma of HIV type 1-positive patients is correlated with the HIV type 1 RNA titers. AIDS Res Hum Retroviruses 23:391–397. doi:10.1089/aid.2006.0124. 17411372

[35] Ferrucci A, Nonnemacher MR, Wigdahl B. 2013. Extracellular HIV-1 viral protein R affects astrocytic glyceraldehyde 3-phosphate dehydrogenase activity and neuronal survival. J Neurovirol 19:239–253. doi:10.1007/s13365-013-0170-1. 23728617 PMC3709860

[36] Agarwal N, Iyer D, Patel SG, Sekhar RV, Phillips TM, Schubert U, Oplt T, Buras ED, Samson SL, Couturier J, Lewis DE, Rodriguez-Barradas MC, Jahoor F, Kino T, Kopp JB, Balasubramanyam A. 2013. HIV-1 Vpr induces adipose dysfunction in vivo through reciprocal effects on PPAR/GR co-regulation. Sci Transl Med 5:213ra164. doi:10.1126/scitranslmed.3007148. PMC4009012 24285483

[37] Levy DN, Refaeli Y, Weiner DB. 1995. Extracellular Vpr protein increases cellular permissiveness to human immunodeficiency virus replication and reactivates virus from latency. J Virol 69:1243–1252. doi:10.1128/JVI.69.2.1243-1252.1995. 7815499 PMC188697

[38] Gangwani MR, Kumar A. 2015. Multiple protein kinases via activation of transcription factors NF-kappaB, AP-1 and C/EBP-delta regulate the IL-6/IL-8 production by HIV-1 Vpr in astrocytes. PLoS One 10:e0135633. doi:10.1371/journal.pone.0135633. 26270987 PMC4535882

[39] Cheng X, Cheng X, Mukhtar M, Acheampong EA, Srinivasan A, Rafi M, Pomerantz RJ, Parveen Z. 2007. HIV-1 Vpr potently induces programmed cell death in the CNS in vivo. DNA Cell Biol 26:116–131. doi:10.1089/dna.2006.0541. 17328670

[40] Torres L, Noel RJ. Jr. 2014. Astrocytic expression of HIV-1 viral protein R in the hippocampus causes chromatolysis, synaptic loss and memory impairment. J Neuroinflammation 11:53. doi:10.1186/1742-2094-11-53. 24655810 PMC3994341

[41] Chen M, Dong Y, Simard JM. 2003. Functional coupling between sulfonylurea receptor type 1 and a nonselective cation channel in reactive astrocytes from adult rat brain. J Neurosci 23:8568–8577. doi:10.1523/JNEUROSCI.23-24-08568.2003. 13679426 PMC6740373

[42] Pergakis M, Badjatia N, Chaturvedi S, Cronin CA, Kimberly WT, Sheth KN, Simard JM. 2019. BIIB093 (IV glibenclamide): an investigational compound for the prevention and treatment of severe cerebral edema. Expert Opin Invest Drugs 28:1031–1040. doi:10.1080/13543784.2019.1681967. PMC6893098 31623469

[43] Caffes N, Kurland DB, Gerzanich V, Simard JM. 2015. Glibenclamide for the treatment of ischemic and hemorrhagic stroke. Int J Mol Sci 16:4973–4984. doi:10.3390/ijms16034973. 25749474 PMC4394459

[44] Khanna A, Walcott BP, Kahle KT, Simard JM. 2014. Effect of glibenclamide on the prevention of secondary brain injury following ischemic stroke in humans. Neurosurg Focus 36:E11. doi:10.3171/2013.10.FOCUS13404. PMC4234034 24380477

[45] Sheth KN, Simard JM, Elm J, Kronenberg G, Kunte H, Kimberly WT. 2016. Human data supporting glyburide in ischemic stroke. Acta Neurochir Suppl 121:13–18. doi:10.1007/978-3-319-18497-5_3. 26463916 PMC5189719

[46] Li G, Bos S, Tsetsarkin KA, Pletnev AG, Despres P, Gadea G, Zhao RY. 2019. The roles of prM-E proteins in historical and epidemic Zika virus-mediated infection and neurocytotoxicity. Viruses 11:157. doi:10.3390/v11020157. PMC6409645 30769824

[47] Kopp JB, Klotman ME, Adler SH, Bruggeman LA, Dickie P, Marinos NJ, Eckhaus M, Bryant JL, Notkins AL, Klotman PE. 1992. Progressive glomerulosclerosis and enhanced renal accumulation of basement membrane components in mice transgenic for human immunodeficiency virus type 1 genes. Proc Natl Acad Sci U S A 89:1577–1581. doi:10.1073/pnas.89.5.1577. 1542649 PMC48495

[48] Zhao RY, Liang D, Li G, Larrimore CW, Mirkin BL. 2010. Anti-cancer effect of HIV-1 viral protein R on doxorubicin resistant neuroblastoma. PLoS One 5:e11466. doi:10.1371/journal.pone.0011466. 20628645 PMC2898807

[49] Li G, Park HU, Liang D, Zhao RY. 2010. Cell cycle G2/M arrest through an S phase-dependent mechanism by HIV-1 viral protein R. Retrovirology 7:59. doi:10.1186/1742-4690-7-59. 20609246 PMC2909154

[50] Yardan T, Erenler AK, Baydin A, Aydin K, Cokluk C. 2011. Usefulness of S100B protein in neurological disorders. J Pak Med Assoc 61:276–281. 21465945

[51] Mehta RI, Tosun C, Ivanova S, Tsymbalyuk N, Famakin BM, Kwon MS, Castellani RJ, Gerzanich V, Simard JM. 2015. Sur1-Trpm4 cation channel expression in human cerebral infarcts. J Neuropathol Exp Neurol 74:835–849. doi:10.1097/NEN.0000000000000223. 26172285 PMC4620032

